# Express Attentional Re-Engagement but Delayed Entry into Consciousness Following Invalid Spatial Cues in Visual Search

**DOI:** 10.1371/journal.pone.0003967

**Published:** 2008-12-17

**Authors:** Benoit Brisson, Pierre Jolicœur

**Affiliations:** Centre de Recherche en Neuropsychologie et Cognition, Université de Montréal, Montréal, Québec, Canada; Harvard Medical School, United States of America

## Abstract

**Background:**

In predictive spatial cueing studies, reaction times (RT) are shorter for targets appearing at cued locations (valid trials) than at other locations (invalid trials). An increase in the amplitude of early P1 and/or N1 event-related potential (ERP) components is also present for items appearing at cued locations, reflecting early attentional sensory gain control mechanisms. However, it is still unknown at which stage in the processing stream these early amplitude effects are translated into latency effects.

**Methodology/Principal Findings:**

Here, we measured the latency of two ERP components, the N2pc and the sustained posterior contralateral negativity (SPCN), to evaluate whether visual selection (as indexed by the N2pc) and visual-short term memory processes (as indexed by the SPCN) are delayed in invalid trials compared to valid trials. The P1 was larger contralateral to the cued side, indicating that attention was deployed to the cued location prior to the target onset. Despite these early amplitude effects, the N2pc onset latency was unaffected by cue validity, indicating an express, quasi-instantaneous re-engagement of attention in invalid trials. In contrast, latency effects were observed for the SPCN, and these were correlated to the RT effect.

**Conclusions/Significance:**

Results show that latency differences that could explain the RT cueing effects must occur after visual selection processes giving rise to the N2pc, but at or before transfer in visual short-term memory, as reflected by the SPCN, at least in discrimination tasks in which the target is presented concurrently with at least one distractor. Given that the SPCN was previously associated to conscious report, these results further show that entry into consciousness is delayed following invalid cues.

## Introduction

Visual-spatial attention can be deployed covertly to specific locations in space in absence of head or eye movements. Voluntary deployments of covert attention have been studied extensively using predictive spatial cueing paradigms [1]. In predictive spatial cueing experiments, trials begin with either a central or a peripheral cue, followed by a target, to which a speeded response is often required. In most trials the target appears at the cued location (valid trials), but on a minority of trials it appears at another location (invalid trials). It is well established that reaction times (RT) are shorter when the target appears at the cued location (in valid trials) than when it appears at an uncued location (in invalid trials). The stage(s) of target processing that are modulated by predictive cues have been strongly debated. Several results, such as the interaction of target luminance with cue validity [2] provided support for an early selection hypothesis, which proposes that the cueing RT effect can be explained by attention-related perceptual facilitation. However, other results, such as the absence of a cueing effect on detection sensitivity accompanied by a lower decision criterion in valid trials [3] supported a late, post-perceptual interpretation of the cueing RT effect, which proposes that cue information does not affect perceptual processes, but rather biases the participants' decision criterion for emitting a response [4].

Event-related potentials (ERPs), which provide continuous millisecond-by-millisecond measures of distinct covert cognitive processes interposed between the stimulus onset and the overt response, have provided valuable insights in this debate. Indeed, an enhancement of the early occipital P1 (90–130 ms) and/or N1 (150–200 ms) components of the ERP are typically observed in predictive spatial cueing paradigms [5], [6]. Given that these early amplitude modulations have been observed for both relevant and irrelevant information presented at an attended location [7]–[9], and that they seem to arise in early extrastriate visual areas, usually without (or with very little) latency or scalp distribution modulations [10], it has been postulated that they reflect sensory-perceptual gain control mechanism that amplify signals at attended locations [11]. It is important to note that although both P1 and N1 attentional modulations are often observed together, important dissociations of these two effects have been reported, indicating that they may well reflect different mechanisms. For example, while the P1 effect has been observed both in discrimination and detection tasks, the N1 effect seems to be specifically tied to discrimination tasks [6]. Also, when a large portion of bilateral displays were used, as in the present study, an enhanced P1 contralateral to the attended item was accompanied by a larger N1 ipsilateral to the attended item [12], [13].

Although it is now commonly accepted that visual-spatial attention enhances sensory-perceptual processing, probably through gain control mechanisms, it is not clear where in the processing stream processes are in fact accelerated in valid compared to invalid trials. In other words, where in the processing stream do the early P1/N1 amplitude effects translate into faster processing? This is an important question because processing must, at some point, be accelerated in order to produce the observed cueing effects on RT.

The present study was designed to constrain the locus of the cueing RT effect by measuring the latencies of two lateralised ERP components: the N2pc (N2 *posterior contralateral*) [14]–[16] and the sustained posterior contralateral negativity (SPCN) [17]–[23].

The N2pc is thought to reflect visual-spatial attention mechanisms that separate relevant and irrelevant perceptual information in bilateral, multi-element search arrays (a mechanism hereafter referred to as visual selection). The N2pc, which typically starts about 180 ms post-target onset and lasts about 100 ms, is maximal at posterior electrode sites contralateral to an attended item and is isolated by subtracting activity at electrode sites ipsilateral to the attended item from the corresponding activity at electrode sites contralateral to the attended item (e.g., PO7/PO8). Luck and colleagues, who were the first to study this component meticulously in visual search tasks, suggested that the N2pc reflects distractor suppression processes [15], [16]. Others, who have used bilateral displays with only one distractor, have argued that the N2pc reflected target enhancement processes [14]. Nonetheless, even if there is still an ongoing debate on the specific processes that underlie the N2pc, it is widely accepted as a valid index of visual selective attention (for a review, see [24]).

The SPCN, which follows the N2pc in the contralateral minus ipsilateral difference wave, is thought to reflect visual short-term memory activity [18]–[23]. One major finding that links the SPCN to visual short-term memory is that the amplitude of the SPCN, which is sustained throughout the retention interval, increases as the number of to-be-remembered items in the visual display increases, but only up to the participants' visual short-term memory capacity [22], [23]. The increase of SPCN amplitude as the number of to-be-remembered items increases has also been reported in choice tasks that were not memory tasks *per se* [18]. In this last study, a modulation of the SPCN amplitude by memory load was not accompanied by a modulation of the N2pc. Combined with a complementary dissociation obtained in previous dual-task experiments (i.e., an attenuation of the N2pc and a delay of the SPCN onset latency without any modulation of the SPCN ultimate amplitude [17], [25]), the results show a double dissociation of the N2pc and SPCN, strongly suggesting that the SPCN is not merely a prolongation of the N2pc, but that the N2pc and SPCN are indeed two functionally distinct components (see also [26]). In agreement with this view, it has been demonstrated that while the N2pc was present for both speeded detection and discrimination tasks, the SPCN was only present in the latter [27]. As the authors have noted, these results further support the proposal that fine analysis of a visual item requires an active maintenance of visual information in VSTM until a decision is made [28].

Interestingly, the SPCN amplitude has been correlated to conscious report. For example, it has been demonstrated that delayed-offset four dot masking (also called object-substitution masking [29]), which reduces report accuracy of the masked item, does not attenuate the N2pc, but seems to have a large effect on the SPCN [30]. The reduction of the SPCN amplitude associated to a reduction in report accuracy has also been observed in the attentional blink paradigm, where dual-task interference is reflected by both a decrease in second target report accuracy and a sharp attenuation of the SPCN elicited by the second target [19], [20], [31]. In contrast, in the psychological refractory period paradigm, where dual-task interference is usually reflected by an increase in RT to the second target without any effect on second target report accuracy, the SPCN onset latency was lengthened as dual-task interference increased, but finally reached a similar amplitude in all conditions, in contrast to the N2pc, which was attenuated, but not delayed [17], [25]. As in the previously mentioned experiments, the SPCN amplitude seemed to follow closely report accuracy, and suggests that the time at which a visual representation is encoded in a format that supports conscious report can be tracked by measuring the SPCN onset latency.

Given that the N2pc is linked to visual selection and the SPCN is linked to visual short-term memory activity (and conscious report), it is possible, by measuring both the N2pc and SPCN onsets in the context of a spatial cueing paradigm, to evaluate whether the cueing effect on RTs where accounted for, at least in part, by the acceleration of processes at or before visual selection, by the acceleration of processes interposed between visual selection and the transfer into visual short-term memory, or by an acceleration of processes after the transfer into visual-short term memory.

In addition, the N2pc results can provide important information as to the time required to shift attention from one location in space to another. Indeed, several theories in attention research presume, more or less explicitly, that the three operations underlying shifts of attention (e.g., disengagement, movement, and re-engagement) take time, and that the time required to accomplish these operations can account for a multitude of attentional phenomena. In visual search, for example, models such as the feature integration theory [32] claimed that attention needs to shift from item to item when the target is defined by conjunctions of features, producing increasing RT functions with set size in these conditions. In the inhibition of return (IOR) literature, an influential theory related the notion of IOR with foraging in visual search [33], [34], implying that attention moves from item to item, and that IOR reduces the probability of returning to a previously inspected location. Classic studies of object-based attention also suggest that attention takes time to shift from attended to unattended locations and/or objects [35], and time-consuming shifts of attention have been assumed more or less explicitly to underlie the costs observed when attention is captured by sudden onsets [36], [37]. Several papers in the contingent capture literature also imply that attention is drawn to the distractor location, and then has to return to the target location [38], [39]. However, it is difficult to tell from behavioural results whether it is the shift of attention per se that takes time, or what takes place downstream, after attention has re-engaged at the new location. On the other hand, differences in N2pc onset latency between valid and invalid conditions could be taken as a direct measure of the time required to disengage attention from one location, move it to a new location, and re-engage at that new location, and help evaluate the timecourse of attentional shifts with more precision.

One important challenge in measuring ERPs in experiments with multiple events is to eliminate all confounding overlapping brain activity. By subtracting the ipsilateral from the contralateral waveforms, it is possible to isolate the N2pc and SPCN from all activity that is not lateralized with respect to the side of presentation of the target, such as sensory and response activity in the present study (for a discussion, see [17]). However, this subtraction does not eliminate attentional activity related to the cued side (e.g., P1/N1 cueing effect). It was therefore crucial to estimate and subtract this activity, especially given the fact that it superimposes itself to the N2pc/SPCN with opposite polarity in valid trials (where the target appears at the cued side) and invalid trials (where target and cued side are opposite). This was accomplished by including “no-target” trials in which the cue was followed by a bilateral visual display containing two distractors instead of a target and a distractor. The no-target trials enabled us to obtain an estimate of the attentional activity caused by the cue in absence of any target-related N2pc/SPCN. ERPs obtained in no-target trials were then subtracted from ERPs obtained in both valid and invalid trials. This subtraction method depends on the validity of the assumption that the ERPs in the no-target trial are identical to the ERPs in the valid and invalid trials, except for the absence of the N2pc and SPCN. Given that no-target trials are in fact no-go trials, it is true that they should elicit a larger N2 at fronto-central sites [40]–[42] and a larger and more anterior P3 [41], [43]. However, given that difference between go and no-go ERPs, which have been linked to response inhibition and/or conflict resolution, are not lateralized in respect to the side of presentation of the target, they will be eliminated by the subtraction of the ipsilateral from the contralateral waveform, and therefore do not compromise the validity of our subtraction procedure (for other successful uses of difference waves, see [25], [44]–[46]).

## Methods

### Participants

Twenty-two volunteers participated in this experiment for pay (25 $ Canadian) after signing a written informed consent document. Six participants had to be excluded from the analyses (see below). Thus 16 participants (ages 19–35, mean age 22.9 years, 11 female) remained in the sample. All were neurologically intact and reported having normal or corrected-to-normal visual acuity and color vision. The study protocol was vetted by the appropriate ethics committee at the Université de Montréal.

### Stimuli and procedure

Participants sat in a dimly lit, electrically shielded room, facing a computer screen, at a viewing distance of 57 cm. The experiment comprised one practice block of 40 trials followed by 16 experimental blocks of 80 trials.

**Figure 1:**
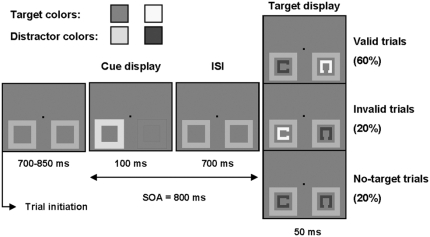
Stimulus sequence in valid, invalid, and no-target trials. One four-alternative discrimination speeded response was required on each trial (except in no-target trials) as to the location of the gap in the target-colored square. Colors were equiluminant red, green, blue and yellow in the actual experiment. The target-colors were counterbalanced between participants. In this example, target-colors are red and yellow.

Each trial was initiated by pressing the “N” and “V” keys simultaneously with the right and left index fingers respectively. Figure 1 illustrates the sequence of events in a trial. A fixation point appeared at the center of the computer screen along with two gray placeholder boxes. The fixation point and the placeholders were visible throughout the remainder of the trial. The placeholders subtended a visual angle of 2°×2° and their centre was 1° below and 3.5° to the left or right of fixation. A brief 100 ms color change of the placeholders, 700 ms to 850 ms after trial initiation, acted as the cue display. One placeholder changed to a target-color and the other to a distractor-color. The colors were red, blue, green, and yellow. All four colors and gray were approximately equiluminant to equate low sensory responses and were presented on a dark background. The target colors were blue and red for four participants, yellow and green for four participants, red and yellow for four participants, and blue and green for the remaining four participants. The target-colored placeholder, which indicated the most probable location of the upcoming target, appeared randomly to the left and right of fixation and appeared randomly in each of the two possible target colors.

In 80% of all trials, the cue display was followed by a 50 ms target display containing one target and one distractor, each appearing in the center of one placeholder. Seventy-five percent of these trials were valid trials, in which the target appeared in the target-colored placeholder, and 25% were invalid trials, in which the target appeared in the distractor-colored placeholder. The cue-target stimulus onset asynchrony (SOA) was 800 ms. The target and the distractor were colored squares, each with a gap in one side (different for each square). Both squares in the target display subtended a visual angle of 1°×1° and the gaps were 0.33°. The colors used in the target display were always different than the colors used in the cue display, so that a color change was present at both the target location and the distractor location for both valid and invalid trials. A speeded four-choice response was required on each trial, as to the location of the gap in the target-colored square. Response keys were “C,” “V,” “N,” and “M” for left, bottom, up, and right gaps, respectively. Participants pressed the “C” and “V” keys with the middle and index fingers of the left hand, and the “N” and “M” keys with the index and middle fingers of the right hand. Instructions emphasized the importance to respond as quickly and accurately as possible, and participants were informed to take into account the cue to maximize general performance.

In the remaining 20% of all trials, the target display was replaced by a distractor display that contained two distractors. The two distractors were in the same distractor color, which was chosen to be different from the distractor color in the cue display, so that a color change was also present at both locations in these “no-target” trials. As mentioned in the introduction, no-target trials were used to estimate the cue-related attention effects (e.g., P1/N1 effects), in absence of any target induced N2pc/SPCN. No responses were required in these trials.

Both target and no-target trials ended with the simultaneous disappearance of the fixation point and placeholder boxes, and appearance of a visual feedback at fixation, 1250 to 1750 ms after the response (or after distractor display onset in no-target trials). A “+” or “−” indicated a correct or incorrect response, respectively. Participants were instructed to maintain central eye fixation throughout the trial and blink only when the feedback was on the screen.

### Electrophysiological recording and analysis

The electroencephalogram (EEG) was recorded from 64 active Ag/AgCl electrodes (BioSemi ActiveTwo system) mounted on an elastic cap and referenced to the average of the left and right mastoids. Electrodes were placed according to the International 10/10 system. The horizontal electrooculogram (HEOG), recorded as the voltage difference between electrodes placed lateral to the external canthi, was used to measure horizontal eye movements. The vertical electrooculogram (VEOG), recorded as the voltage difference between two electrodes placed above and below the left eye, was used to detect eye blinks. A low-pass filter of 40 Hz was applied and the EEG and EOG signals, digitized at 256 Hz, were averaged offline.

Trials with eye blinks (VEOG>80 µV), large horizontal eye movements (HEOG>30 µV), and/or artefacts at electrode sites of interest (i.e., >80 µV at PO7 and/or PO8 electrode sites) were rejected. Moreover, only trials with a correct response between 100 and 1200 ms were analysed. Six participants were excluded because more then half of the trials in at least one condition (no-target trials, invalid trials, and/or valid trials) were rejected. None of the remaining participants had residual eye movements that deviated more than 3.3 µV (i.e., corresponding to about 0.2° of visual angle) after rejection criteria were applied [47]. The HEOG criteria was lowered to 25 µV for one participant, to 23 µV for one participant, and to 20 µV for two more participants so that the residual HEOG would be less than 3.3 µV.

The ERPs were computed by averaging the EEG starting 100 ms prior to the target display onset and ending 600 ms post-target display onset, and baseline corrected based on the 100 ms pre-target display period.

Ipsilateral and contralateral waveforms were computed separately for valid and invalid trials, as well as for no-target trials. When analysing the P1/N1 effects, laterality was defined with respect to the cued side, whereas in the N2pc and SPCN analyses, laterality was defined with respect to the side of presentation of the target. In the latter analyses, to remove all activity that was not lateralized with respect to the target location (such as sensory and response related activity), contralateral minus ipsilateral waveforms were computed. Furthermore, to isolate the N2pc and SPCN from the preceding P1/N1 cueing effect, the no-target ERPs were subtracted from both the valid and the invalid ERPs. N2pc and SPCN amplitude and latency measures were obtained from these corrected difference waves.

Latency measures were obtained with the jackknife method [48]–[50]. With the jackknife method, N grand average waveforms are computed with N-1 participants (a different participant is removed for each waveform). Latency measures are obtained for each of these n grand average waveforms, and the values are submitted to a conventional analysis of variance (ANOVA), but for which the F-values must be adjusted according to
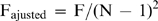
(see [50] for a general proof of this adjustment).

Moreover, in order to perform correlations between possible component latency effects and the RT effect, we recovered the N2pc and SPCN onsets of each participant in each condition based on the jackknife values using the following formula:

Let L_i_ be the latency of the component for subject i and L_GA_ be the latency of the grand average waveform that includes all subjects, which we can represent as the average of the individual subject waveform latencies as follows:
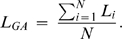



Let L_Jack_ be the latency of the grand average jackknife waveform that excludes subject j: 
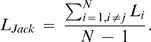



From these two values, we can recover the latency of the waveform for subject i, L_i_ as follows: 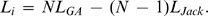



## Results

### Behavioral results

Only trials with correct responses and reaction times between 100 ms and 1200 ms were included in the reaction time (RT) analyses, and outliers were excluded using the method described in Van Selst and Jolicœur [51]. As expected, shorter RTs were produced in valid (685 ms) than in invalid trials (714 ms; *F*(1, 15) = 16.04, *p*<.002). As is often the case with highly visible, unmasked targets, no validity effects was observed on accuracy (95.5 % in valid trials and 94.9% in invalid trials, *F*(1, 15) = 1.29, *p*>.27). Mean percentage of false alarms in no-target trials was 1.13%.

### Electrophysiological results

#### P1/N1 cueing effects

**Figure 2:**
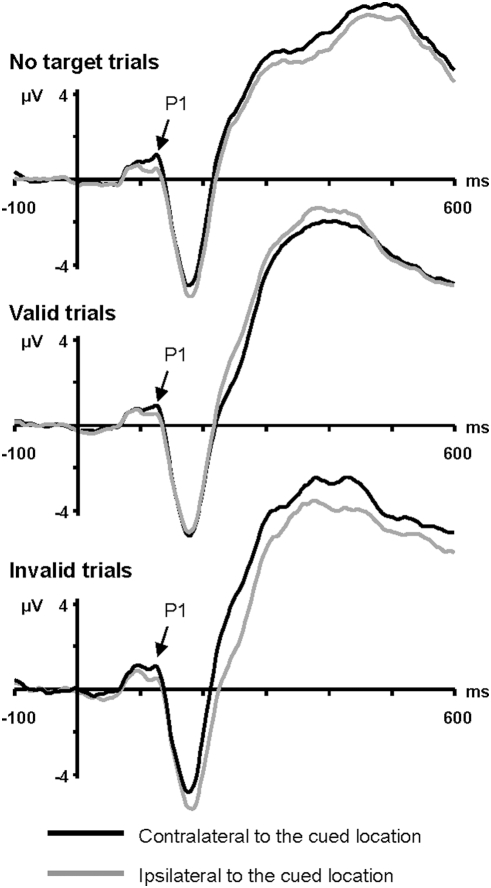
Grand-average event-related potential (ERP) waveforms time-locked to the target display onset at PO7 and PO8 sites for valid, invalid, and no-target trials. Contralateral and ipsilateral were defined in relation to the cued location.

Mean amplitude of the P1 (90–130 ms) and N1 (150–200 ms) were analysed at PO7 and PO8 sites. P1 measurements were submitted to repeated measures ANOVAs in which validity (no target vs. valid vs. invalid) and laterality (ipsilateral vs. contralateral) were included as within-subject factors. Ipsilateral and contralateral waveforms were defined with respect to cued side, where attention should have been at target display onset. These waveforms are shown in Figure 2. A main effect of laterality (*F*(1, 15) = 17.77; *p*<.001) indicated that the P1 was larger contralateral (0.91 µV) than ipsilateral (0.55 µV) to the attended side. No main effect of validity (*F*<1) nor laterality×validity interaction (*F*(2, 30) = 1.04; *p*>.36) were present, indicating that the no-target condition provides a good estimate of cue related attentional activity in absence of any target related N2pc/SPCN activity.

Given that the N1 time-window overlaps with the N2pc, N1 laterality effects, uncontaminated by the N2pc, could only be measured in the no-target condition. A main effect of laterality (*F*(1, 15) = 9.80; *p*<.007) indicated that the N1 was larger ipsilateral (−4.41 µV) than contralateral (−4.02 µV) to the attended location, as observed in previous studies using bilateral target displays [12], [13].

#### N2pc

**Figure 3:**
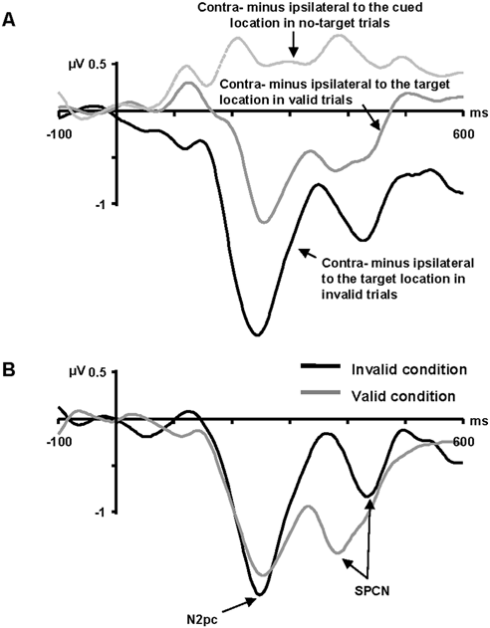
A) Contralateral minus ipsilateral difference waves time-locked to target display onset at PO7/PO8 sites in valid, invalid, and no-target trials. Contralateral and ipsilateral were defined relative to the cued location in no-target trials, but were redefined in respect to the target location in valid and invalid trials. B) Contralateral minus ipsilateral corrected difference waves for valid and invalid trials. The corrected valid difference wave was obtained by subtracting the no-target difference wave from the valid difference wave, whereas the invalid corrected difference wave was obtained by summing the no-target difference wave and invalid difference wave. Shown are the 10 Hz low-pass filtered waveforms.

To isolate the N2pc and SPCN, corrected difference waves were computed following three steps. First, the ipsilateral and contralateral waveforms were redefined with respect to the location of the target, but only in target present trials (remember that contralateral and ipsilateral were defined with respect to the cued location in Figure 2). Second, ipsilateral waveforms were subtracted from the contralateral waveforms in each type of trial, leading to the contralateral minus ipsilateral difference waves presented in Figure 3 (panel A). It is important to note here that in Figure 3A, ipsilateral and contralateral waveforms in no-target trials are still defined with respect to the cued location. In the final step, the no-target difference wave was subtracted from the valid difference wave (because the target was presented at the cued location) and was summed to the invalid difference wave (because the target was presented opposite to the cued location). The resulting contralateral minus ipsilateral corrected difference waves, from which we obtained the N2pc and SPCN measures, are presented in Figure 3 (panel B).

**Figure 4:**
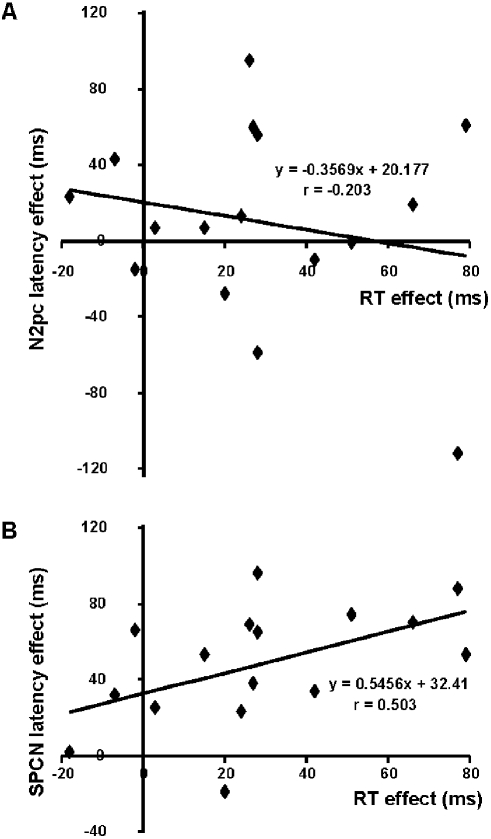
Scatterplots of the A) N2pc latency effect and RT effect, and of the B) SPCN latency effect and RT effect. Individual N2pc and SPCN latencies were recovered from the jackknife values according to the formula presented in the Methods section.

Mean amplitude of the N2pc (210–290 ms), measured at PO7/PO8 sites, was similar in the valid (−1.52 µV) and invalid conditions (−1.60 µV; *F*<1). To assess possible latency effects, an additional 10 Hz low-pass filter was applied to reduce noise (and thus increase robustness) and the time at which the corrected difference waves reached −0.6 µV, starting at 140 ms post-target onset, was measured using the jackknife method [48]–[50]. As suggested in Figure 3B, this analysis revealed no hint of any effect of cue validity on N2pc latency (valid condition = 181 ms, invalid condition = 187 ms; *F_adjusted_*<1). Performing the analysis on the original 40 Hz low-pass filtered waveform also yielded a non significant result (*F_adjusted_*<1). Using the formula presented in the Method section, we recovered individual N2pc latency values from the jackknife values and then performed a two-tailed Pearson correlation between the N2pc latency effect and the RT effect. As expected from the absence of a cue validity effect on N2pc latency, the correlation was not significant (r = −.203, *p*>.45). A scatterplot of the N2pc latency effect and the RT effect is presented in Figure 4 (panel A).

**Figure 5:**
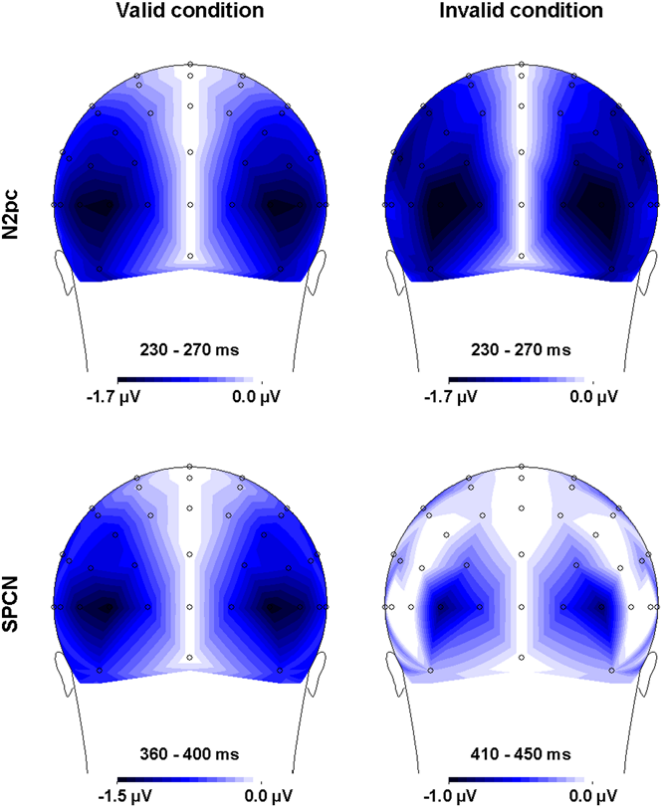
Scalp distributions of the electrical potentials measured during the N2pc (230–270 ms for both the valid and invalid conditions) and SPCN (360–400 ms in the valid condition and 410–450 ms in the invalid condition) time windows. The scalp distributions were calculated on the basis of the corrected contralateral minus ipsilateral difference waves used to calculate the N2pc and SPCN, and are thus symmetrical about the midline.

The scalp distributions of the N2pc are shown in Figure 5 (upper panels) for both the valid and invalid conditions. To evaluate whether the N2pc scalp distributions were typical and similar between conditions, we normalized the activity centered on the component's peak (mean amplitude in the 230–270 ms time-window in both validity conditions) according to the procedure described by McCarthy and Wood [52], and posterior electrode pairs (O1/O2, PO3/PO4, PO7/PO8, P1/P2, P3/P4, P5/P6, P7/P8, P9/P10) were submitted as a within-subject factor in an ANOVA in which the second within-subject factor was validity (valid vs. invalid). A Greenhouse-Geisser correction was used for the estimation of *F* statistics. A main effect of electrode (*F*(7, 105) = 4.79; *p*<.01) indicated that the N2pc amplitude was different across these electrodes and was maximal at PO7/PO8 sites. No main effect of validity was observed (*F*<1), and the absence of any validity×electrode interaction (*F*<1) suggests similar scalp distributions in the valid and invalid conditions.

#### SPCN

The SPCN is the second negative deflection in the contralateral minus ipsilateral difference wave (see Figure 3B). Mean amplitude in the 350–400 ms time range at PO7/PO8 sites was larger in the valid condition (−1.40 µV) than in the invalid condition (−0.23 µV; *F*(1, 15) = 5.24; *p*<.04). No main effect of validity was present in the later 410–460 ms time range (valid condition = −1.01 µV, invalid trials = −0.79 µV; *F*<1). Importantly, to determine whether the apparent shift in SPCN latency was statistically reliable, we applied a 10 Hz low-pass filter and used the jackknife method. Given that the SPCN follows the N2pc, an earlier onset of the former means a greater overlap with the latter. In this situation, the SPCN is thus superimposed on the greater negativity of the N2pc, modulating its amplitude. In order to minimized the variability caused by differences in N2pc amplitude at the onset of the SPCN, we did not use a fixed amplitude criterion to measure the SPCN latency, as was done for the N2pc, but instead we measured the time at which the SPCN, in each N-1 waveform, reached half of its maximum (peak amplitude) minus minimum (the junction between N2pc and SPCN) amplitude. As suggested in Figure 3B, the SPCN latency occurred earlier in the valid condition (355 ms) than in the invalid condition (400 ms; *F_adjusted_*(1, 15) = 32.89; *p*<.0001). Performing the analysis on the original 40 Hz low-pass filtered waveform did not change the significance of the result (*F_adjusted_*(1, 15) = 33.04; *p*<.0001). An analysis based on peak amplitude latency also revealed a main effect of validity (*F*(1, 15) = 16.10, *p*>.001).

The positive deflection in the SPCN time range in the no-target difference wave (see Figure 3A) could have potentially increased the SPCN latency difference between valid and invalid conditions. To test this possibility, we further filtered the no-target difference wave with a 3 Hz low-pass filter before correcting the valid and invalid difference waves. Despite the fact that the severe filtering of the no-target difference wave practically eliminated the positive deflection in the SPCN time range, the difference in SPCN latency was still significant between valid (355 ms) and invalid conditions (392 ms; *F_adjusted_*(1, 15) = 7.07; *p*<.018), indicating that the SPCN latency difference was not artificially created by the subtraction method.

Using the recovered SPCN latency values, we performed a two-tailed Pearson correlation between the SPCN latency effect and the RT effect. Importantly, the correlation was significant (r = .503, *p*<.047). A scatterplot of the SPCN latency effect and the RT effect is shown in Figure 4 (panel B).

The scalp distributions of the SPCN are shown in Figure 5 (lower panels) for both the valid and invalid conditions. As for the N2pc, we evaluated whether the scalp distributions of the SPCN were typical and similar between conditions by normalizing the activity centered on the component's peak (mean amplitude in the 360–400 ms time-window in the valid condition and in the 410–450 ms time-window in the invalid condition) according to the procedure described by McCarthy and Wood [52], and posterior electrode pairs (O1/O2, PO3/PO4, PO7/PO8, P1/P2, P3/P4, P5/P6, P7/P8, P9/P10) were submitted as a within-subject factor in an ANOVA in which the second within-subject factor was validity (valid vs. invalid). A Greenhouse-Geisser correction was used for the estimation of *F* statistics. A main effect of electrode (*F*(7, 105) = 3.16; *p*<.02) indicated that the SPCN was, as in previous reports [17], [18], [53], different across these electrodes and maximal at PO7/PO8 sites. No main effect of validity was observed (*F*(1, 15) = 2.85; *p*>.11). Importantly, the absence of any validity×electrode interaction (*F*<1) suggests similar scalp distributions in the valid and invalid conditions.

## Discussion

The locus of the spatial cueing RT effect was investigated by measuring the onset latency of two lateralized ERP components: the N2pc, an index of visual selective attention, and the SPCN, an index of visual-short term memory. The first important result was that both the onset and the amplitude of the N2pc were unaffected by cue validity, despite the fact that a larger P1 amplitude was observed contralateral to the attended (cued) location. The absence of any cueing effect on the N2pc is important for several reasons. First, it replicates previous findings [54] and confirms that the N2pc is not related to the shift of attention *per se*, but rather to visual selection. Indeed, if the N2pc reflected (at least in part) the shift of attention to the target location, then its amplitude should have been attenuated in valid trials relative to invalid trials, since attention should have been at the correct location at target onset in valid trials, and therefore should not need to shift again in these trials, contrary to invalid trials. Second, the N2pc results demonstrate for the first time that although there was a clear benefit for targets presented at the attended location rather than at the unattended location, in terms of mean RT, the amplitude change in the P1 component apparently does not translate in an immediate acceleration of visual target selection, as indexed by the N2pc, at least in conditions were target luminance is high, and the selection cue is a pop-out color, as in this study. Indeed, it has been demonstrated that both P1 amplitude and N2pc latency were sensitive to target luminance [55]. Therefore, it still needs to be tested whether sensory gain, as reflected by the P1 cueing effect, results in an N2pc latency shift in low target luminance conditions. Nevertheless, the present results demonstrate that RT effects can occur without N2pc latency effects, suggesting that the “spotlight” of attention, if there is such a thing, can move very quickly, as thought it has no mass [56]–[58]. Also, the N2pc results further reaffirms the notion that targets can pop-out of the visual display in the sense that attention can jump to the appropriate location very quickly under appropriate conditions. Finally, given that target colors and distractor colors were equiluminant, and counterbalanced across subjects, the fact that attention can find the target location just as fast in invalid trials as in valid trials, is consistent with recent work on contingent attentional capture, which suggests that a critical component of attentional control lies in top-down control settings that can bias how incoming signals are amplitude-modulated, gated, and processed from very early to later stages of processing [59]–[63].

In contrast to the N2pc, the SPCN, which had a typical scalp distribution in both validity conditions, occurred earlier in valid trials. Furthermore a positive correlation was observed between cueing effects on SPCN latency and on RTs, suggesting that latency differences that contribute to the predictive cueing RT effect occur at (or before) transfer into visual short-term memory, where visual information is encoded in a format that supports conscious report [17], [19], [20], [30], [31], and is thought to be actively maintained in order to perform fine analyses [27], [28]. Previous studies have addressed directly the question of whether the SPCN and N2pc are functionally distinct [18], [26], [27], [53], and all concluded that, although the scalp topography of these components are very similar, they index distinct cognitive processes (i.e., N2pc indexes visual selective attention and SPCN indexes VSTM retention activity). The present results provide an additional dissociation that further demonstrates that the N2pc and SPCN are functionally distinct.

It is unlikely that the effect of cue validity on SPCN onset latency was the consequence of differences in N2pc parameters, such as timing variability or offset, which could have led to down stream effects on the SPCN. Indeed, increased variability in the timing of a component is reflected by what is termed “component smearing”. Smearing is characterized by a decrease in the amplitude and an increase in the duration of the component (see [47] for a detailed explanation). Results show that the amplitude of the N2pc was not significantly different across valid and invalid conditions. Although it is very difficult to measure N2pc offset in this experiment, do to the overlap of the SPCN, Figure 3 suggests that if there was a difference in the offset of the N2pc, it would tend to occur later in the valid condition. Because there was no difference in N2pc onset, a later offset would also mean increased duration of the component in the valid condition. Therefore, if there were any differences in overall variability, even though significant differences in N2pc amplitude were not detected, the variability would be larger (and the N2pc offset would be later) in the valid than in the invalid condition. Logically, greater variability and later offsets could lead to an apparent increase in the onset latency of a following component, but not the contrary. Therefore, if it is true that the variability and/or the offset of the N2pc increased in the valid compared to the invalid condition, and if these differences led to apparent downstream effects on the SPCN, then we would predict that the SPCN, if anything, would occur earlier in the invalid condition, which is opposite to what has been observed.

Although transfer in visual short-term memory occurs relatively late in the processing stream, it still takes place before decision making. The present results therefore confirm that RT effects in predictive cueing studies are not solely du to the biasing of the participants decision criterion, as a purely late selection theory would postulate. However, the present results do not rule out the possibility that late decision related processes also contribute to the RT effect.

### Conclusion

The modulation of the P1 amplitude as a function of predictive spatial cueing provides good evidence that attention had been deployed at the cued location (whether valid or invalid). The fact that the latency of the N2pc was the same for valid and invalid trials suggests strongly that attention could be re-deployed quasi-instantaneously, in invalid trials, to the target location following the appearance of the target in the visual display, and therefore that the time required to move attention could not explain the RT cueing effect. However, the delay of the SPCN onset latency in invalid trials reveal that following the re-deployment of attention, the information flow into visual-short term memory, and thus entry into consciousness, is delayed when the target appears at an unattended location. In sum, the present results strongly suggest that although predictive spatial cueing affects the amplitude of early ERP components, such as the occipital P1, the stage(s) of processing in which such early amplitude effects are translated into latency differences that could explain the observed RT effects must occur after visual selection processes giving rise to the N2pc, but at or before transfer in visual short-term memory, as reflected by the SPCN.
